# Correction: Beliefs, attitudes and practices towards scabies in central Ghana

**DOI:** 10.1371/journal.pntd.0011313

**Published:** 2023-05-05

**Authors:** The PLOS Negelected Tropical Diseases Staff

The organization that funded this work was inadvertently left out. We have added the below funder.

This work was financially supported by NWA Idea Generator (grant number NWA.1228.192.144). The funder had no role in study design, data collection and analysis, decision to publish, or preparation of the manuscript.
