# Supplementary material for: Beliefs, attitudes and practices towards scabies in central Ghana
Source: PLoS Negl Trop Dis. 2023 Feb 22;17(2):e0011175. doi: 10.1371/journal.pntd.0011175 (PMC9987785; doi:10.1371/journal.pntd.0011175)
Supplement: Attachment — Submitted filename: REVIEW COMMENTS PTND.docx [file pntd.0011175.s001.docx]

1. A more detailed description of the region/area under study: say a brief of the health system and economic setup for the area, the total population if possible so we know what 128 participants is the proportion of for the sake of readers not familiar with Ghanaian societies would be key in my opinion. If walking in the street for two months gets you 30 people with present of past scabies, this could be considered too little for a heavily endemic area or indeed a lot for a less endemic socially upper class area where scabies is rare.
2. L136/6: How was the targeted recruitment number determined?
3. For past scabies patients, how long ago were you to have been diagnosed with scabies to be included in the study? Is anyone, including a 70 year old who recalls having had scabies when he was 20 years old vs a 22 year old who had scabies last week be eligible? This information would be important as most of the information is collected via recalling from past events and one has to remember to do this. In addition, as perceptions change, it would be interesting to know how people giving their opinion on what they experienced when they had scabies 20 years ago still compare to that whose diagnosis was a fortnight ago before the study started in 2020, for instance.
4. Is ‘*duration for participants to find out their symptoms were scabies’* synonymous with *‘duration between onset of scabies complaints till a visit to health centre to seek care’*? (L 170/5), if not is data available on the former group about when they sought care after realizing they had scabies? This may be of interest to a reader to understand the delays and their factors better.
5. From line (L) 176 to 207, several related but different terms have been used and one wonders whether they are referring to the same thing. The title talks about perceived ‘*causes*’, then there is mention of other terms as follows: factors ‘*predisposing to scabies*’, *practices* ‘*contributing*’ to scabies, *behaviours ‘linked’ to* scabies, ‘*risk*’ of scabies, factors ‘*leading’ to scabies*, ‘*associating*’ the disease with scabies and *risk behaviours*. May I suggest if the title of that section be changed from ‘causes’ to a more general term to supporting the fact that the paragraph is describing a diverse perceived etiological factors of the disease (hence the different terms used) rather than just a ‘cause’ or better still, presenting using a uniform term?
6. L286, please revisit sentence to remove the repeated word
7. I am not able to tell how the following information was collected or study related procedures were made, kindly elaborate please:
i. How was delay in seeking care for scabies among *past* scabies patients collected?
ii. In the community, where patients were recruited on the street as they walked in their village (close to and hence they could invite the study team into their homes but also may be away from their houses), or some probably just passing by from their own other village to another destination, please elaborate how the patients were examined in private? (L141/1)
iii. In the community, it appears knowledge about whether you ever had scabies or not in the past was required to allow one to be assigned appropriate group. If we take *non scabies* group and past scabies subset of the *scabies* group, how confident are the study team that the respondents were accurate with diagnosis about their previous scabies or no scabies history given such lack of knowledge about the disease in this community explored in the latter sections of the manuscript? I would expect at least a reference to their medical records to prove that history, at least, which is a challenge if they meet and recruit people in the street.
iv. L131 to 134: says the study team arrived and met the CBSV at a public gathering and went down a randomly selected street to recruit people. Was this the practice every day that during the September to November 2020 data collection period i.e they would arrive at the same public meeting place, which had several outlet distributary streets, on a daily basis, then select any different random street and follow it for that day or they had different public places and different streets but in the same region or it was the same street that was chosen on first day that they went down along for the entire study duration starting from the same point and time consistently? Or may be a different scenario from the three I was trying to imagine.
v. A good part of the discussion and conclusion hinges on the beliefs of the respondents about the disease (witchcraft and curse). The numbers are quiet small though (about 7 out of 128 respondents) were of this view. Don’t you think it could be misleading to consider these responses representative enough to warrant such bold statements that this community had poor beliefs? More so when according to figure 1, there are higher percentages for responses like *parasite, microorganism, friends/family you have, environment* etc which may be closer to the truth than witchcraft/curse but are totally ignored in the discussion while the negative but minute points in this case are being emphasised on?
vi. I note that there is presentation of only those results that the authors have chosen to discuss at the end without allowing the readers to have a view of both opinions. For example, only the reasons for longer delay to seek care by community participants (mean delay of 30 days) are described yet there was a delay of 14 days in those sampled from the clinic whose reasons are not highlighted (L 232/42). For seeking care in formal settings, only the reasons for such among clinic recruits (73 %) are discussed yet 50 % of those in the community sought care in formal facilities too (L 253). It would be nice to hear from the authors why they have chosen this way of presenting results, if they think discussing the contrasting views is still not appropriate.
