# Supplementary material for: Beliefs, attitudes and practices towards scabies in central Ghana
Source: PLoS Negl Trop Dis. 2023 Feb 22;17(2):e0011175. doi: 10.1371/journal.pntd.0011175 (PMC9987785; doi:10.1371/journal.pntd.0011175)
Supplement: Attachment — Submitted filename: Review comments for PNTD.docx [file pntd.0011175.s002.docx]

**Review comments**

- What clinical criteria was used to diagnose scabies?
- How were participants with active scabies at recruitment clinically identified. What diagnostic criteria were used to identify/diagnose scabies?
- The authors should include the cost in US dollars highlighted below

**The following grammatical corrections were noted**.

Line 63: predominantly affects persons of low socioeconomic living in rural communities with limited

Line 162, ….of participants in was 32.3

Line 194: These finding were confirmed the data obtained in the open questions. Sentence needs to be corrected

Line 253: the dermatology clinic were more likely to seek…….

Line 261: The one person who visited a traditional health spent 30 Ghana cedis (~$ 6 US dollars).

Line 275: …per national guidelines. This should be referenced

Line 307: other neglected tropical diseases of the skin in the West Africa….

Line 332: treat (if treatment is available)

Line 357: Indeed, in a previous study in on

Line 389: There is the need to increased community awareness of

Line 395: Solomon Islands, scabies has found

Line 408: this is the first study that assess beliefs, attitudes and practices toward

Line 411: …seeking for as part of integrated control for neglected tropical diseases in Ghana.
