# Supplementary material for: Beliefs, attitudes and practices towards scabies in central Ghana
Source: PLoS Negl Trop Dis. 2023 Feb 22;17(2):e0011175. doi: 10.1371/journal.pntd.0011175 (PMC9987785; doi:10.1371/journal.pntd.0011175)
Supplement: Attachment — Submitted filename: Response to reviewer comments_19012023.docx [file pntd.0011175.s003.docx]

26^th^ January 2023

Kumasi Centre for Collaborative Research

Kwame Nkrumah University of Science and Technology

Kumasi, Ghana

The Editor

PLOS Neglected Tropical Diseases

Dear Sir,

**Re: Decision on your PLOS Neglected Tropical Diseases submission (PNTD-D-22-01409) - [EMID:1815af1d13d56242]: ‘’Beliefs, attitudes and practices towards scabies in central Ghana’’**

My co-authors, Lotte Suzanne van Rietschoten, Michael Ntiamoah Oppong, Kwabena Oppong Amoako, Kabiru Mohammed Abass, Bernard Akoto Anim, Dennis Odai Laryea, Richard Odame Phillips, Ymkje Stienstra and I, wish to thank the editors and reviewers for the feedback on our manuscript.

Our study provides data to guide the development of context specific interventions for scabies control in central Ghana. We highlight the need to enhance health education to promote early care seeking, enhance knowledge of communities on impact and dispel negative perceptions about scabies in Ghana.

My co-authors and I have taken note of the review comment and revised the manuscript as suggested. The comments we believe have improved our manuscript. We wish to submit the revised manuscript for publication in your esteemed journal. In the attached reply, the reviewer questions are in blue font and our responses are in red font.

We look forward to the further review of the manuscript and the subsequent publication of same.

We count on your usual kind assurances.

Yours Sincerely,

Dr Yaw Ampem Amoako

Corresponding author

**Point-by-point responses to review comments**

Reviewer's Responses to Questions
Thank you to the authors for submitting this manuscript, which is of general public health importance as it is the first study to attempt to assess beliefs, attitudes and practices towards scabies in Ghana. The paper is also of interest as it indicated that some people seemed unaware that treatment for scabies is free under the national health insurance scheme in Ghana which highlights the need for educational programmes to promote the availability of free and effective treatment for persons with health insurance. There are some significant concerns, however, regarding the methodology and conclusions from the results. These are outlined in detail by reviewer number 2 and each of these points need to be addressed prior to any re-submission.

Response: We thank you for the comment emphasizing the manuscript to be of public health importance. We have tried to address the concerns in our responses below.

Additional minor comment:

Fig 4. Does this reflect the data from only the scabies group? If so, please amend the legend title accordingly.

Response: We changed the legend of Fig 4 accordingly. It now reads *’Fig 4 Facilitators of care seeking in the formal health system by participants in the scabies group’.*

Reviewer's Responses to Questions

Key Review Criteria Required for Acceptance?

Reviewer #1: an excellent and relevant study which was well conducted

Reviewer #2: Not clear how the sample size was reached and how the actual recruitments took place.

Response: See detailed response to question 2 below

Reviewer #3: The objective of the study is clearly articulated though there is no hypothesis stated. The study design is appropriate for the objective. There are no ethical concerns regarding this study.

Does the analysis presented match the analysis plan?

Reviewer #1: well analysed and presented

Reviewer #2: No comment

Reviewer #3: The analysis presented corresponds to the analysis plan and the results are clearly and completely presented and the Figures are clear.

Are the conclusions supported by the data presented?

Reviewer #1: Conclusions are supported and there are recommendations stemming from this work

Reviewer #2: I do not think the conclusions are fair reflection of the findings. Details in the attachment.

Response: We address the reviewers’ comments (see responses to questions [7(v) – 7(vi)] below

Reviewer #3: The conclusions support data represented and the limitations are mentioned. The data and its usefulness understanding the topic are discussed as well as its public health relevance in development of community control programs and recommendation for enhancement of health education in Ghana and availability of scabies treatment by the Government

Use  this section for editorial suggestions as well as relatively minor modifications of existing data that would enhance clarity. If the only modifications needed are minor and/or editorial, you may wish to recommend “Minor Revision” or “Accept”.

Reviewer #1: Accept

Reviewer #2: (No Response)

Reviewer #3: Overall, the paper is well written and there are few grammatical edits to be made in addition to the details of the clinical diagnostic criteria of scabies in this study.

Summary and General Comments

Reviewer #1: An important study on a common and distressing condition in communities. Understanding  underlying belief systems and providing insights into the cause is key to encouraging earlier presentation and improving the quality of life of those affected

Reviewer #2: Possible bias in the interpretation of the result where the authors have a message they want to convey and totally disregard the data at their disposal but follow faint leads to their conclusions.

Response: This comment is linked to the reviewer’s comments on the conclusion not reflecting the findings and we hope our answers below [questions 7(v) – 7(vi)] sufficiently address the reviewer’s worries

Reviewer #3: This paper reports on the Beliefs, attitudes, and practices towards scabies in central Ghana. It presents important findings that are relevant in understanding the problem in the community and how to tackle them to ensure scabies control. The discussion is relevant and extensive and covers the topic appropriately.

**Review comments**

- What clinical criteria was used to diagnose scabies?
- How were participants with active scabies at recruitment clinically identified. What diagnostic criteria were used to identify/diagnose scabies?

Response: This has been clarified in the methods section by inserting the following sentences: ‘’For participants with active scabies, the diagnosis was made based on a history of pruritus and the typical appearance and distribution of the rash and/ or the presence of burrows. For participants with past scabies, the diagnosis was based on a self-reported history of disease in the preceding 1 year.’’ (Line 149-152)

- The authors should include the cost in US dollars highlighted below

Response: Done as suggested (line 282)

**The following grammatical corrections were noted**.

Line 63: predominantly affects persons of low socioeconomic living in rural communities with limited

Response: Revised. Sentence now reads: ‘’…and the disease predominantly affects persons of low socioeconomic status living in rural communities with limited access to healthcare (line 63)

Line 162, ….of participants in was 32.3

Response: Revised. The word ‘’in’’ has been deleted (line 179)

Line 194: These finding were confirmed the data obtained in the open questions. Sentence needs to be corrected

Response: Corrected. The word ‘’by’’ has been inserted to make the sentence clearer (line 214)

Line 253: the dermatology clinic were more likely to seek…….

Response: Done as suggested (line 274)

Line 261: The one person who visited a traditional health spent 30 Ghana cedis (~$ 6 US dollars).

Response: Done as suggested (line 282)

Line 275: …per national guidelines. This should be referenced

Response: The reference to the national guidelines (#24) was already made in the methods section. We did not find it appropriate to re-do so in the results section of the manuscript.

Line 307: other neglected tropical diseases of the skin in the West Africa….

Response: We have deleted ‘’the’’ from the sentence (line 329)

Line 332: treat (if treatment is available)

Response: We have inserted ‘’is’’ as suggested (line 355)

Line 357: Indeed, in a previous study in on

Response: We have deleted ‘’in’’ from the sentence (line 380)

Line 389: There is the need to increased community awareness of

Response: Section has been corrected appropriately (line 412)

Line 395: Solomon Islands, scabies has found

Response: We have replaced ‘’has’’ with ‘’was’’ (line 418)

Line 408: this is the first study that assess beliefs, attitudes and practices toward

Response: Revised. Sentence now reads ‘’ Despite the limitations, this is the first study that has assessed beliefs, attitudes and practices towards scabies in Ghana.’’ (Line 438)

Line 411: …seeking for as part of integrated control for neglected tropical diseases in Ghana.

Response: Revised. We have deleted the word ‘’for’’ (line 441)

Reviewer 2

1. A more detailed description of the region/area under study: say a brief of the health system and economic setup for the area, the total population if possible so we know what 128 participants is the proportion of for the sake of readers not familiar with Ghanaian societies would be key in my opinion. If walking in the street for two months gets you 30 people with present of past scabies, this could be considered too little for a heavily endemic area or indeed a lot for a less endemic socially upper class area where scabies is rare.

Response: We have done as suggested by adding the following to the section titled study settings (line 104-113)

‘The Asante Akim North district is one of the forty-three districts in Ashanti region and has a population of 68,186 according to the 2010 Ghana Population and Housing Census. About 53.5 percent of the population is rural. Males constitute 48.8 percent and females represent 51.2 percent. The district has relatively youthful population with slightly more than half (50.6%) of the population below 20 years. The average household size in the district is 4.5 persons per household. Of the population 11 years and above, 79.2% are literate. Majority of the employed population, (61%) are engaged in agriculture (mainly farming and fishing) while 16.8% are service and sales workers. The Agogo Presbyterian Hospital is the only secondary health facility in the district which receives referrals from health centres and other primary level facilities within the district [21].’’

We agree it to be useful to have background information on the Asante Akim North district, but the reviewer indicates wanting to know the proportion of the 128 participants in relation to the total population. This would be relevant if the study attempts to estimate the economic burden or the prevalence/incidence of scabies which were not amongst the objectives of the study.

2. L136/6: How was the targeted recruitment number determined?

Response: To clarify, we have added the following sentences in the methods section (line 140-143): ‘’The sample size was intended to both generate information on geographical variation and differences between scabies patients and controls. Furthermore, data saturation in the open questions was included in the decision making on the sample size.’’

3. For past scabies patients, how long ago were you to have been diagnosed with scabies to be included in the study? Is anyone, including a 70 year old who recalls having had scabies when he was 20 years old vs a 22 year old who had scabies last week be eligible? This information would be important as most of the information is collected via recalling from past events and one has to remember to do this. In addition, as perceptions change, it would be interesting to know how people giving their opinion on what they experienced when they had scabies 20 years ago still compare to that whose diagnosis was a fortnight ago before the study started in 2020, for instance.

Response: Based on this useful comment we realize this was not well described in the manuscript. Participants in the scabies group were only included if that scabies was clinically evident in the past year. We have revised to reflect that participants in the scabies group either had scabies at the time of the interview or scabies in the past year. The section now reads ‘For participants with past scabies, the diagnosis was based on a self-reported history of clinically evident disease in the preceding 1 year.’’ (Line 151-152). We also added how long ago the scabies started in the scabies group [median (IQR) 45 (27 – 90)] days in line 185 of the results section.

4. Is ‘*duration for participants to find out their symptoms were scabies’* synonymous with *‘duration between onset of scabies complaints till a visit to health centre to seek care’*? (L 170/5), if not is data available on the former group about when they sought care after realizing they had scabies? This may be of interest to a reader to understand the delays and their factors better.

Response: Individuals with scabies may have had the diagnosis suggested by family members, teachers, or community health volunteers. The ‘’ *median duration for participants to find out their symptoms were due to scabies’* refers to when this suggestion was first made. However, the ‘’*visit to the health centre to seek care’’* may only have occurred later. This fact is reflected in the differences in the interquartile ranges for the two measures of delay.

5. From line (L) 176 to 207, several related but different terms have been used and one wonders whether they are referring to the same thing. The title talks about perceived ‘*causes*’, then there is mention of other terms as follows: factors ‘*predisposing to scabies*’, *practices* ‘*contributing*’ to scabies, *behaviours ‘linked’ to* scabies, ‘*risk*’ of scabies, factors ‘*leading’ to scabies*, ‘*associating*’ the disease with scabies and *risk behaviours*. May I suggest if the title of that section be changed from ‘causes’ to a more general term to supporting the fact that the paragraph is describing a diverse perceived etiological factors of the disease (hence the different terms used) rather than just a ‘cause’ or better still, presenting using a uniform term?

Response: We have taken the reviewer’s suggestion and changed the title of the section to ‘’Perceived aetiological factors of scabies’’ (line 193)

6. L286, please revisit sentence to remove the repeated word

Response: Done

7. I am not able to tell how the following information was collected or study related procedures were made, kindly elaborate please:
i. How was delay in seeking care for scabies among *past* scabies patients collected?

Response: Delay in seeking care was assessed using the median (IQR) time from onset of scabies complaints till a visit to health centre to seek care. In response to review comment 3 above, we added that participants either currently had scabies or had scabies in the past year. For such an itching experience, we believe participants were still able to recall the delay in seeking care.

ii. In the community, where patients were recruited on the street as they walked in their village (close to and hence they could invite the study team into their homes but also may be away from their houses), or some probably just passing by from their own other village to another destination, please elaborate how the patients were examined in private? (L141/1)

Response: Once participants in the community expressed their willingness to partake, they were led to the community centre where the clinical examinations were performed. The methods section has been revised appropriately to reflect this and the section now reads: ‘’Participants from the community were asked if they had any active rash following which the rash was examined by one of the trained members of the team to diagnose scabies. All clinical examinations were conducted in private (in the clinic or community centre as appropriate) to ensure confidentiality.’’ (Line 157-159)

iii. In the community, it appears knowledge about whether you ever had scabies or not in the past was required to allow one to be assigned appropriate group. If we take *non scabies* group and past scabies subset of the *scabies* group, how confident are the study team that the respondents were accurate with diagnosis about their previous scabies or no scabies history given such lack of knowledge about the disease in this community explored in the latter sections of the manuscript? I would expect at least a reference to their medical records to prove that history, at least, which is a challenge if they meet and recruit people in the street.

Response: We have rephrased to indicate that we included only participants with a clinical diagnosis of scabies < 1 year ago as we recognise that information about a more recent diagnosis (< 1 year) is more reliable than information about a diagnosis from long ago. Further, we had previously highlighted this in the limitation section by stating that: ‘’ The diagnosis of scabies by community members who reported having scabies in the past year may have been inaccurate. However, since the aim of the study was to assess ideas on scabies, we included participants who thought they had scabies and not only persons with diagnostically confirmed scabies.’’ (line 423-424)

Additionally, we have added to the limitations the following sentences: ‘’There might have been a recall bias by participants regarding a past history of scabies. We sought to limit this by including only individuals with a past diagnosis <1 year ago as we believe that information about a more recent diagnosis is more reliable than that about a diagnosis made long ago. A good proof about a history of scabies may be obtained from the medical records of participants. However, we were unable to access the medical records of participants with past scabies recruited from the community setting. It was only possible to refer to the records of those scabies participants recruited from the dermatology clinic.’’ (Line 426-433)

iv. L131 to 134: says the study team arrived and met the CBSV at a public gathering and went down a randomly selected street to recruit people. Was this the practice every day that during the September to November 2020 data collection period i.e they would arrive at the same public meeting place, which had several outlet distributary streets, on a daily basis, then select any different random street and follow it for that day or they had different public places and different streets but in the same region or it was the same street that was chosen on first day that they went down along for the entire study duration starting from the same point and time consistently? Or may be a different scenario from the three I was trying to imagine.

Response: The communities involved are small rural villages which typically have a single major road. This was the route that the team walked to recruit the participants.

v. A good part of the discussion and conclusion hinges on the beliefs of the respondents about the disease (witchcraft and curse). The numbers are quiet small though (about 7 out of 128 respondents) were of this view. Don’t you think it could be misleading to consider these responses representative enough to warrant such bold statements that this community had poor beliefs? More so when according to figure 1, there are higher percentages for responses like *parasite, microorganism, friends/family you have, environment* etc which may be closer to the truth than witchcraft/curse but are totally ignored in the discussion while the negative but minute points in this case are being emphasised on?

Response: We have presented all the data on the perceived aetiologies of scabies in the results section. Because of the potential of scabies becoming a community outbreak following delayed diagnosis and treatment (*even for a few cases*), any misconception that can impact early diagnosis and initiation of appropriate care seeking ought to be taken seriously and adequately addressed by (country) control programmes. A belief in supernatural cause of disease has been associated with delay in health seeking (*see references 7, 22, 24, 29, 30 and 31 of this manuscript*). A thinking that scabies is related to the water people drink may lead to unnecessary changes in behaviour. Designing and implementing a control programme for scabies requires an understanding of the local context / country specific issues that have the potential to hamper early diagnosis, appropriate health seeking and early treatment of the disease. Further, context specific issues should be taken into account when designing interventions and educational messages. We have highlighted the misconceptions about scabies in this study even while recognising actual known causes were quite often mentioned.

We have added the following sentence to the discussion section: ‘’An appreciable proportion of participants reported scabies to be caused by a parasite, microorganism, or ‘’friends/family you have’’. (Line 332-334)

We also added to the conclusion the sentence (line 444-445):

‘’Although actual known causes of scabies were quite often mentioned by participants, there were several misconceptions about scabies.’’

Education on scabies by control programmes will have to focus on changing the misconceptions while emphasizing the positive knowledge and attitudes about the disease within Ghana.

vi. I note that there is presentation of only those results that the authors have chosen to discuss at the end without allowing the readers to have a view of both opinions. For example, only the reasons for longer delay to seek care by community participants (mean delay of 30 days) are described yet there was a delay of 14 days in those sampled from the clinic whose reasons are not highlighted (L 232/42). For seeking care in formal settings, only the reasons for such among clinic recruits (73 %) are discussed yet 50 % of those in the community sought care in formal facilities too (L 253). It would be nice to hear from the authors why they have chosen this way of presenting results, if they think discussing the contrasting views is still not appropriate.

Response: The reasons for delay are not only included for the community setting. The reasons for the delayed care seeking described represents the views of all persons in the scabies group (both those recruited from clinic and community setting). We tried to further clarify this by modifying the title of figure 3 (*Fig 3 Reasons for delayed care seeking among scabies group (community setting and dermatology clinic)*. See line 264-265

Similarly in line 291-292, Fig 4 shows the *‘’facilitators of care seeking in the formal health system by participants in the scabies group’’ (this includes both scabies participants from community and clinic setting).* Further, we have included in the results section the following sentences: ‘Within the scabies group, the disease was mentioned to be due to a parasite (13.4% vs 23.9%) or a small organism (14.9% vs 20.9%) and friends/ family you have (23.9% vs 26.9%) respectively for participants recruited from the dermatology clinic and community settings (figure 2).’’ Line 203-206
